# Contrastive Learning-Driven Image Dehazing with Multi-Scale Feature Fusion and Hybrid Attention Mechanism

**DOI:** 10.3390/jimaging11090290

**Published:** 2025-08-26

**Authors:** Huazhong Zhang, Jiaozhuo Wang, Xiaoguang Tu, Zhiyi Niu, Yu Wang

**Affiliations:** 1 College of Aviation Electronic and Electrical Engineering, Civil Aviation Flight University of China, Chengdu 641450, China; 2 Sichuan Province Engineering Technology Research Center of General Aircraft Maintenance, Civil Aviation Flight University of China, Guanghan 618307, China

**Keywords:** image dehazing, hybrid attention mechanism, multi-scale dynamic feature fusion, InfoNCE loss function

## Abstract

Image dehazing is critical for visual enhancement and a wide range of computer vision applications. Despite significant advancements, challenges remain in preserving fine details and adapting to diverse, non-uniformly degraded scenes. To address these issues, we propose a novel image dehazing method that introduces a contrastive learning framework, enhanced by the InfoNCE loss, to improve model robustness. In this framework, hazy images are treated as negative samples and their clear counterparts as positive samples. By optimizing the InfoNCE loss, the model is trained to maximize the similarity between positive pairs and minimize that between negative pairs, thereby improving its ability to distinguish haze artifacts from intrinsic scene features and better preserving the structural integrity of images. In addition to contrastive learning, our method integrates a multi-scale dynamic feature fusion with a hybrid attention mechanism. Specifically, we introduce dynamically adjustable frequency band filters and refine the hybrid attention module to more effectively capture fine-grained, cross-scale image details. Extensive experiments on the RESIDE-6K and RS-Haze datasets demonstrate that our approach outperforms most existing methods, offering a promising solution for practical image dehazing applications.

## 1. Introduction

Under complex atmospheric conditions, haze not only significantly degrades the visual quality of images but also negatively impacts various computer vision tasks such as object detection and image segmentation [1]. Effectively removing haze and restoring clear visual information has thus become a critical issue in the fields of computer vision and image processing.

Current dehazing methods can be broadly categorized into two types: traditional prior-based methods and deep learning-based methods. Traditional methods, such as Dark Channel Prior (DCP) [2] and Color Attenuation Prior (CAP) [3], rely on atmospheric scattering models [4,5]. While they perform well in some simple scenarios, they often fail in more complex scenes, leading to loss of high-frequency details and reduced image clarity.

In recent years, deep learning methods, particularly the application of Convolutional Neural Networks (CNNs) [6], have significantly enhanced the performance of image dehazing. Deep learning methods automatically learn features, offering stronger adaptability in handling complex environments. In natural scenes, haze is typically unevenly distributed, and with variations in haze density, lighting conditions, and scene complexity, the effectiveness of dehazing methods also varies. To address this issue, this paper introduces contrastive learning [7], an important paradigm in deep learning. The core idea of contrastive learning is to minimize the distance between positive sample pairs (similar images) while maximizing the distance between negative sample pairs (dissimilar images) [8]. In the dehazing task, we treat hazy images as negative samples and their corresponding clear images as positive samples. By employing contrastive learning, the model can better distinguish inherent scene features from distortions caused by haze, thereby enhancing its ability to understand the image content. This enables the model to retain the scene’s structure and texture information while effectively removing haze, resulting in more accurate and visually consistent dehazing outcomes.

Despite the significant progress deep learning-based methods have made in dehazing performance, challenges still remain in detail restoration and high-frequency information handling. Particularly when processing image edges or complex backgrounds, existing methods often struggle to simultaneously restore global features and local details. To overcome these challenges, this paper introduces two innovative techniques: a multi-scale dynamic feature fusion method and a hybrid attention mechanism.

First, to address the blurring or distortion issues that arise in existing methods when handling high-frequency details, this paper proposes the multi-scale dynamic feature fusion method. This method dynamically adjusts the frequency bands of filters to ensure that fine-grained details are captured effectively at different scales. Specifically, the cascaded dynamic filtering process integrates features from multiple scales, preserving high-frequency details while effectively handling complex backgrounds and detail-rich images. This method significantly enhances the model’s ability to restore details, particularly in image edges and complex backgrounds, reducing the loss of important information [9].

Second, the hybrid attention mechanism integrates multiple attention strategies, allowing the model to dynamically adjust its focus [10]. This mechanism aggregates information at different layers, enabling the network to flexibly allocate computational resources based on different regions and feature dimensions in the image. As a result, it can restore local details while maintaining the integrity of global information. Especially in regions severely affected by haze, the model can pay more attention to these areas, improving the accuracy and naturalness of the dehazing effect [11].

By combining the multi-scale dynamic feature fusion method and the hybrid attention mechanism, the proposed model can more accurately restore areas severely affected by haze, preserving more details and significantly improving the dehazing effect.

Finally, to further enhance the model’s ability to capture global features, this paper introduces the Transformer architecture [12], utilizing self-attention mechanisms to model long-range dependencies. The Transformer architecture, which has achieved significant success in natural language processing, has also shown superior performance in image processing tasks, effectively capturing global information and further enhancing the dehazing effect [13].

Our proposed method, Contrastive Learning-driven image dehazing with Multi-scale feature Fusion and Hybrid Attention mechanism, is abbreviated as CL-MFHA. The contributions of CL-MFHA can be summarized as follows.

- In this paper, we introduce contrastive learning as an innovative approach to image dehazing, enabling the model to effectively preserve the structure and texture of the scene while removing haze, thereby achieving more accurate and visually consistent dehazing results.
- We propose a multi-scale dynamic feature fusion strategy integrated into the Transformer-enhanced architecture and a hybrid attention mechanism, which together enhance the model’s ability to focus on critical regions and preserve fine-grained details across multiple scales, thereby improving both local and global feature restoration.
- Extensive experiments on popular benchmark datasets, including RESIDE-6K [14], RS-Haze [13], and NH-Haze [15], demonstrate that the proposed method outperforms existing dehazing techniques, delivering superior dehazing accuracy and visual quality.

## 2. Related Work

Early image dehazing methods mainly relied on the atmospheric scattering model, using mathematical techniques to recover true image details [16]. Recently, deep learning-based approaches, such as CNNs [6] and Generative Adversarial Networks (GANs) [17], have gained significant attention. These methods utilize deep learning’s powerful feature extraction and learning capabilities to learn dehazing mappings from large datasets of hazy and clear image pairs, enabling end-to-end dehazing.

The Gated Fusion Network (GFN) [18], proposed by Ren et al., was one of the first deep learning-based methods for image dehazing. It processes the transmission map and atmospheric light separately through two sub-networks then uses a gating mechanism to combine the outputs and recover the clear image. Subsequent models, like AOD-Net [19], have integrated the atmospheric scattering model into CNNs, allowing joint learning of dehazing and underwater image enhancement. However, these models can be limited in extreme weather conditions. Deep residual networks, such as DenseNet [20] and ResNet [21], have improved training stability, feature extraction, and convergence speed, but they require substantial computational resources and can suffer from overfitting.

Some methods combine physical models and deep learning by using deep learning to estimate parameters like transmission and atmospheric light then performing dehazing based on these estimates. For instance, GridDehazeNet [22] uses a grid-based attention mechanism to estimate the transmission map and integrates the atmospheric scattering model, offering high accuracy and adaptability. However, its high computational complexity and reliance on transmission map estimation can limit performance. FFA-Net [10] uses an end-to-end feature fusion attention network to capture multi-scale information, reducing computational cost and improving detail recovery in complex hazy scenes. However, its performance depends on training data diversity and quality, and insufficient data may lead to overfitting or underfitting. Additionally, the single attention mechanism may overly focus on specific regions, neglecting others.

In response, the CL-MFHA model proposed in this paper replaces the single attention mechanism with a hybrid attention mechanism, allowing the model to focus on features at different levels and perform effective information aggregation. This enhances the model’s global context understanding and adaptability to complex scenarios. We also introduce a multi-scale dynamic feature fusion module that expands the receptive field using convolutions with different dilation rates [23], maintaining computational efficiency. This module balances local detail recovery and global context perception, improving the model’s adaptability across different environments.

Another key issue is CNNs’ limited ability to capture long-range dependencies, which restricts global feature extraction. The Transformer architecture addresses this by using self-attention to capture long-range dependencies, enhancing the model’s global context understanding [12]. DehazeFormer [13], a Transformer-based dehazing network, combines Transformer’s global modeling capability with CNNs’ image processing strengths. We integrate Transformer into the CL-MFHA model to enhance its global perception, enabling more natural image restoration and better performance in complex hazy scenes.

In image dehazing tasks, haze distribution is often uneven, particularly in natural scenes where haze density, illumination, and scene complexity vary significantly. To ensure the consistency and realism of dehazed images and enhance adaptability, the model must acquire a deeper semantic understanding. Therefore, we employ contrastive learning by constructing positive and negative sample pairs to optimize the model. Hazy images serve as negative samples, while clear images serve as positive samples, with the objective of minimizing the distance between positive pairs and maximizing the distance between negative pairs in the feature space. This enables the model to distinguish scene features from haze distortions, preserve key details such as textures and edges, and thereby achieve more accurate dehazing [24]. It is important to emphasize that our contrastive learning approach differs significantly from existing works. UCL-Dehaze [24] employs unsupervised contrastive learning to bring recovered images closer to clear images at the global feature level, combined with adversarial training to enhance the realism of generated images. However, it mainly relies on unpaired global feature contrast and pays less attention to local details. AECR-Net [25], on the other hand, introduces hazy images as negative samples in a supervised setting through a lightweight autoencoder structure combined with contrastive regularization, focusing on global consistency but insufficiently modeling fine-grained local features in complex scenes. In contrast, our CL-MFHA model introduces novel designs in both sample construction and augmentation strategies. First, in constructing contrastive pairs, we not only consider the global similarity between restored and clear images but also incorporate multi-scale local feature contrasts, enabling the model to capture fine-grained textures and edge details while maintaining overall consistency. Second, we propose a task-specific data augmentation strategy, including illumination adjustment, color jittering, rotation, and cropping, to generate more diverse positive and negative samples. This significantly enhances the discriminative ability of contrastive learning and improves model generalization. These designs allow CL-MFHA to balance local detail recovery and global consistency in both image enhancement and contrastive learning, resulting in more robust and discriminative feature representations than existing methods.

## 3. The Proposed Method

We now present the details of the proposed method, which are divided into three parts: the overall architecture, the Contrastive Learning-based (CL-based) dehazing, and the Multi-scale dynamic feature Fusion and Hybrid Attention Block (MF-HA-Block).

### 3.1. Overall Architecture

**Figure 1:**
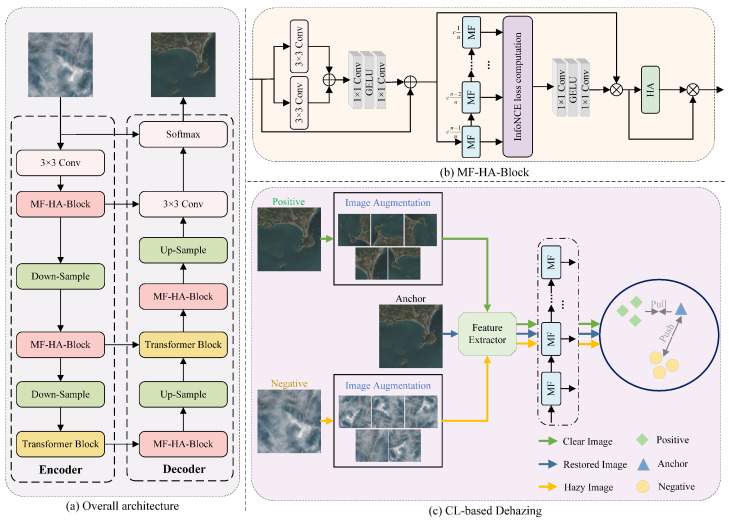
The architecture of the proposed CL-MFHA. (**a**) The overall architecture adopts the popular U-Net structure, where MF-HA-Block is our proposed Multi-scale dynamic feature Fusion and Hybrid Attention Block. (**b**) The proposed MF-HA-Block consists of three processes: the calculation of the InfoNCE loss function (contrastive learning), the Multi-scale dynamic feature Fusion module (MF), and the Hybrid Attention mechanism (HA). (**c**) CL-based dehazing approaches pull the restored image (i.e., the anchor) towards the clear image and push it away from the hazy image.

As can be seen in Figure 1, the proposed model adopts an encoder–decoder architecture. The encoder comprises 3 × 3 convolutional layers, MF-HA-Blocks, Transformer Blocks, and downsampling layers. Correspondingly, the decoder consists of 3 × 3 convolutional layers, MF-HA-Blocks, Transformer Blocks, upsampling layers, and activation function layers. Our network consists of a total of 1130 layers. Each MF-HA-Block contains 7 multi-scale dynamic feature fusion modules and 1 hybrid attention mechanism and incorporates the InfoNCE loss function [26] to guide the contrastive learning process. The InfoNCE loss function models the relationships between positive samples and a large number of negative samples in a high-dimensional feature space, enabling the model to learn more discriminative and robust feature representations at the semantic level. This mechanism not only enhances the separability of feature distributions but also improves the model’s generalization ability and stability across diverse scenarios and complex haze conditions. The overall network architecture leverages contrastive learning principles during training, enabling the model to better retain fine image details and demonstrate enhanced robustness in dehazing tasks. This training process is constrained by the InfoNCE loss function. The multi-scale dynamic feature fusion module expands the receptive field, balancing the perception of global and local information. Then, the hybrid attention mechanism is used to capture more detailed information, further preserving high-frequency features. The method uses a U-Net architecture [27], with skip connections helping the network retain more image feature information during the decoding process, resulting in more accurate output images. To compensate for the limitations of CNNs in capturing global feature information [12], a Transformer Block is introduced in the CL-MFHA model. The Transformer, with 2.03 MB of parameters, is placed in the middle of the network. Through the self-attention mechanism, it captures long-range dependencies and provides richer global information.

### 3.2. Contrastive Learning-Based Dehazing

The construction of positive and negative sample pairs for contrastive learning is based on paired hazy images and their corresponding clear images. The positive sample pair consists of an anchor image and its similar clear image, where the goal for each image pair is to learn the similarity between them through contrastive learning. The negative sample pair consists of the anchor image and its hazy image, with the objective of pushing the anchor image away from the hazy image, helping the model to distinguish features of different scenes. To construct positive and negative sample pairs for contrastive learning, all images undergo a unified series of data augmentation operations, as outlined in [25]. During this process, random cropping is applied, with the cropping position modulated by an edge decay factor, thereby increasing the likelihood of selecting regions near the image boundaries. These edge regions typically exhibit more severe haze and complex interference, thus contributing to greater diversity in local perturbations. To further enrich the structural and distributional variations among samples, random horizontal flipping and rotations at 90-degree intervals are employed. In addition to geometric transformations, we apply illumination adjustment, color jittering, and Gamma correction to both positive and negative sample pairs. These photometric transformations increase the diversity of appearance while preserving semantic content, thereby encouraging the network to focus on essential features rather than superficial variations. Collectively, these augmentation strategies enhance the variability of training data, improve the robustness of feature representations, and boost the model’s generalization performance in complex dehazing scenarios.

**Figure 2:**
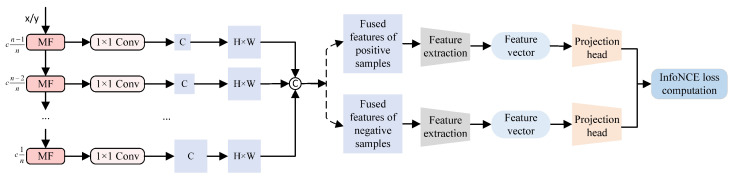
Process of the contrastive learning. The extracted features are input into the MF, where they are unified in terms of both channel dimensions and spatial resolution before being concatenated. The fused features of positive and negative samples, processed in the same manner, are then jointly fed into the InfoNCE loss function for computation.

After augmentation, the images are fed into the network, where intermediate features are extracted through a CNN and Transformer architecture. These features are then uniformly dimension-reduced and concatenated before being input into the projection head, which maps them into the embedding space required for contrastive learning [24]. The training process is constrained by the InfoNCE loss function, which minimizes the feature distance between different augmented versions of the same image while pushing apart representations of different images. This weakens the influence of haze on representation learning and helps construct a well-structured semantic space in the feature domain [28]. The process of the contrastive learning is illustrated in Figure 2. The InfoNCE loss function [26] is defined as:

(1) $$L_{\mathrm{InfoNCE}}=-\log\frac{\exp\left(\frac{\mathrm{sim}(F_{h},F_{c})}{\tau }\right)}{\sum_{j=1}^{M}\exp\left(\frac{\mathrm{sim}(F_{h},F_{j})}{\tau }\right)},$$

where $F_{h}$ denotes the feature vector of the query sample, $F_{c}$ is the feature vector of a positive sample (a sample similar to the query sample), $\mathrm{sim}(F_{h},F_{c})$ indicates the similarity between the query sample and the positive sample, and $\mathrm{sim}(F_{h},F_{i})$ refers to the similarity between the query sample and a set of negative samples (samples that are dissimilar to the query sample). τ is the temperature hyperparameter, and *M* represents the number of negative samples. This loss function optimizes the model by maximizing the similarity between the query sample and the positive sample while minimizing the similarity between the query sample and the negative samples.

### 3.3. Multi-Scale Dynamic Feature Fusion and Hybrid Attention Block

MF-HA-Block is a module that integrates both the Multi-scale dynamic feature Fusion module (MF) and the Hybrid Attention mechanism (HA). Its purpose is to fuse shallow and deep features together, allowing the estimated clear image to retain more details. MF-HA-Block is shown in Figure 1b. In the MF-HA-Block, multiple MFs progressively reduce the number of channels, enabling the model to hierarchically extract features at different scales, thus avoiding information loss due to a single compression step.

#### 3.3.1. Multi-Scale Dynamic Feature Fusion Module (MF)

**Figure 3:**
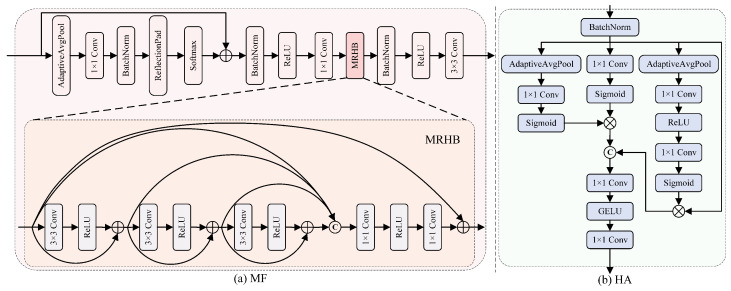
Structure diagram of the multi-scale dynamic feature fusion module and hybrid attention mechanism. (**a**) The MF includes a critical Multi-scale Residual Hybrid Block (MRHB), which retains image details through a unique residual structure. (**b**) The HA incorporates three attention paths that integrate various computational methods to capture information.

The multi-scale dynamic feature fusion module focuses on isolating high-frequency feature maps to restore details such as textures and edges, which are then convolved and fused with the original feature map. The structure of the multi-scale dynamic feature fusion module is shown in Figure 3a. This module uses residual connections to preserve the structural information of the input image, preventing excessive dehazing that could lead to detail loss or image blurring [29].

An important component of the multi-scale dynamic feature fusion module is the Multi-scale Residual Hybrid Block (MRHB). The MRHB contains three 3 × 3 convolutions with different dilation rates, expanding the receptive field while maintaining the same computational cost. The convolution with a low dilation rate focuses on local details, while the convolution with a high dilation rate focuses on large-scale feature information [30]. Finally, two 1 × 1 convolutions are used to integrate the extracted features, resulting in a feature map that combines both local and global information. Therefore, the MRHB can perform two tasks during the dehazing process: restoring local details and enhancing global information perception.

#### 3.3.2. Hybrid Attention Mechanism (HA)

To effectively enhance key features in the dehazing task, a Hybrid Attention mechanism (HA) is designed in the algorithm, as shown in Figure 3b. The HA is primarily based on channel attention [10], integrating different computational methods through multiple attention paths to capture information from different scales. Compared to channel attention, the HA performs global channel modeling, which can more effectively distinguish between regions with thick and thin haze. The module calculates weights using different paths, allowing the network to adaptively adjust the dehazing intensity for different areas. It can enhance the effective features (such as textures, edges, etc.) in thin haze regions while reducing the irrelevant information in thick haze regions. This module uses only 1 × 1 convolutions and pooling, which results in low computational complexity as an auxiliary module, while effectively improving feature representation capability.

### 3.4. Overall Training

The model training process proposed in this paper is designed to ensure that the model can effectively learn both global and local features of images while recovering texture details to a greater extent and overcoming the challenges posed by haze in complex scenes.

Before training begins, we perform data augmentation on the images in the dataset, such as random cropping, rotation, horizontal flipping, illumination adjustment, color jittering, and Gamma correction. Data augmentation increases the diversity of the training data, enhancing the model’s adaptability and robustness in performing dehazing tasks across various scenes. The training process involves four passes through the MF-HA-Block and two passes through the Transformer Block. In the MF-HA-Block, multiple MF modules with progressively reduced channel numbers are applied, followed by uniform dimensionality reduction and concatenation of the intermediate features, which are then mapped to the embedding space required for contrastive learning. The contrastive learning framework optimizes the feature learning process by minimizing the distance between positive sample pairs and maximizing the distance between negative sample pairs. This enables the model to effectively distinguish between haze-induced pseudo-features and inherent scene features in the feature space, with the InfoNCE loss function used to constrain the contrastive learning process. Subsequently, a hybrid attention mechanism is applied, where multiple attention paths flexibly weight feature information, enhancing the model’s expression of high-frequency features, thereby making the dehazed image clearer. The complete U-Net architecture algorithm ultimately generates a model that can effectively handle different haze conditions, recovering images that are not only visually consistent but also more natural while preserving the basic structure and details of the image.

## 4. Experiment

In this section, we present the experimental results of the algorithm and provide an analysis. First, we will demonstrate the model’s performance on multiple datasets and compare it with other state-of-the-art image dehazing models. Then, we will show the results of ablation experiments to evaluate the effectiveness of each model.

### 4.1. Experimental Settings

#### 4.1.1. Implementation Details

The experiments were conducted in a Python 3.12 environment using PyTorch 2.1.0 and implemented on an NVIDIA A800 GPU. The models were evaluated on the RESIDE-6K, RS-Haze, and NH-Haze datasets. For the RESIDE-6K dataset, the training image patch size was set to 256 × 256, with a batch size of 14 and an initial learning rate of $1\times 10^{-5}$, and training was conducted for 1000 epochs. For the RS-Haze dataset, the training image patch size was also 256 × 256, with a batch size of 16 and an initial learning rate of $2\times 10^{-4}$, and training was conducted for 150 epochs. For the NH-Haze dataset, the training image patch size was set to 256 × 256, the batch size was 16, and the initial learning rate was $1\times 10^{-4}$. The network was optimized using the AdamW optimizer. During training, a cosine annealing strategy was employed to adjust the learning rate, gradually reducing it to 1% of its initial value. The proposed CL-MFHA model has a computational complexity of 56.67 GMac, a parameter size of 15.62 MB, and an inference time of 125.35 ms per image.

#### 4.1.2. Datasets

The RESIDE-6K dataset’s training set contains 6000 image pairs, each consisting of a hazy image and a corresponding clear image. All training images are resized to 400 × 400. The hazy images are synthesized by applying a physical model to the clear images, simulating different concentrations and types of haze environments. The dataset includes both indoor and outdoor scenes, covering various environments such as city streets, indoor spaces, natural landscapes, and more. The test set of the RESIDE-6K dataset consists of 1000 image pairs, which are a mix of indoor and outdoor images, and the image size is not adjusted [14]. The RESIDE-6K dataset provides a high-quality benchmark for training and testing image dehazing tasks. The RS-Haze dataset is a publicly available dataset specifically designed for remote sensing image dehazing research. The training set contains 51,300 image pairs, and the test set contains 2700 image pairs. It includes remote sensing images with different levels of haze simulation and is widely used for training and evaluating remote sensing image dehazing algorithms. The RS-Haze dataset is derived from real remote sensing data, covering various geographical scenes such as cities, forests, oceans, and mountainous areas. Each image has varying haze intensity and types, providing diverse training data for dehazing algorithms [13]. The NH-Haze dataset is a high-quality dataset specifically designed for image dehazing tasks in natural haze environments. It contains hazy and clear image pairs captured in real outdoor conditions, covering different lighting conditions, times of day, and weather environments. This dataset provides more challenging natural scene dehazing samples, offering a reliable benchmark for evaluating the performance of dehazing algorithms in real-world scenarios.

#### 4.1.3. Evaluation Metrics

To comprehensively evaluate the performance of the proposed algorithm, three widely used metrics were employed: PSNR, SSIM, and LPIPS. PSNR (Peak Signal-to-Noise Ratio) measures the pixel-wise difference between the restored image and the ground truth, emphasizing low-level reconstruction fidelity. SSIM (Structural Similarity Index Measure) evaluates the structural similarity between images, focusing on perceptual quality and preserving important structural information such as edges and textures. LPIPS (Learned Perceptual Image Patch Similarity) is a deep learning-based perceptual metric that measures visual similarity in a way that aligns better with human perception, capturing differences that PSNR and SSIM may not reflect. By combining these three metrics, we can achieve a comprehensive evaluation of image quality: PSNR for pixel accuracy, SSIM for structural integrity, and LPIPS for perceptual realism. This multi-dimensional assessment ensures a thorough comparison of the dehazing performance across both objective and perceptual aspects.

### 4.2. Quantitative Comparison

We evaluated the performance of the proposed model on the RESIDE-6K and RS-Haze datasets and compared it with state-of-the-art models. All models were trained under the same conditions without using any pretrained models, and both our model and the compared models were trained and tested entirely on the same datasets. The results are shown in Table 1.

GCANet is a traditional CNN-based dehazing method that primarily focuses on local feature extraction from images. Due to its relatively basic network architecture, GCANet exhibits limited dehazing performance, with comparatively lower metrics in both PSNR and SSIM, particularly showing inadequate image quality restoration in complex scenarios. In contrast to GCANet, GridDehazeNet employs a more sophisticated dehazing strategy through grid-based multi-scale feature fusion to enhance cross-level feature interactions. While this approach improves local feature processing to some extent, its grid-structured methodology demonstrates deficiencies in global information integration, achieving only a marginal 0.17 dB PSNR advantage over GCANet on the RS-Haze dataset. FFA-Net, compared to the previous two models, achieves breakthroughs in performance metrics. On the RESIDE-6K dataset, its PSNR reaches 28.32 dB, which is an improvement of 3.26 dB over GridDehazeNet. This significant enhancement is primarily attributed to the feature fusion attention mechanism proposed by this method, which effectively captures both global structures and local details, demonstrating the potential of attention mechanism optimization. DehazeFormer-S further enhances dehazing network performance by leveraging Transformer’s self-attention mechanism to model long-range dependencies, thereby overcoming CNN’s locality constraints. This innovation enables the model to surpass the 30 dB PSNR threshold on RESIDE-6K, outperforming FFA-Net by 2.3 dB. While LKD-Net-B achieves a slightly higher PSNR of 30.67 dB than DehazeFormer-S on RESIDE-6K, its performance is comparatively weaker on RS-Haze. This observation suggests that while LKD-Net-B’s large kernel decomposition technique and dynamic kernel selection mechanism contribute to performance improvement, the method shows limited generalization capability for remote sensing scenarios. ConvIR-B employs a structurally redesigned, multi-branch convolutional architecture with diverse receptive fields, enabling effective modeling of local textures and edge details under non-uniform haze conditions. It ranks second in performance on both the RESIDE-6K and RS-Haze datasets, highlighting its strong capability in handling image dehazing tasks. Our proposed CL-MFHA achieves state-of-the-art performance across all metrics: a PSNR of 31.85 dB, SSIM of 0.980, and LPIPS of 0.014 on RESIDE-6K; and a PSNR of 39.76 dB, SSIM of 0.971, and LPIPS of 0.059 on RS-Haze. Notably, CL-MFHA surpasses the second-best model ConvIR-B by 0.89 dB PSNR on RESIDE-6K and exceeds DehazeFormer-S by 0.19 dB on RS-Haze. These results conclusively demonstrate the significant effectiveness of CL-MFHA in enhancing dehazing performance.

To validate the generalization capability of the dehazing model, we also conducted experiments on the NH-Haze dataset, which contains real haze scenes. The experimental results are shown in Table 2. CL-MFHA still achieves the best performance across all metrics, with a PSNR of 20.98 dB and an SSIM of 0.803. These results demonstrate that CL-MFHA possesses strong generalization ability for dehazing in real-world haze scenarios.

### 4.3. Visual Comparison

**Figure 4:**
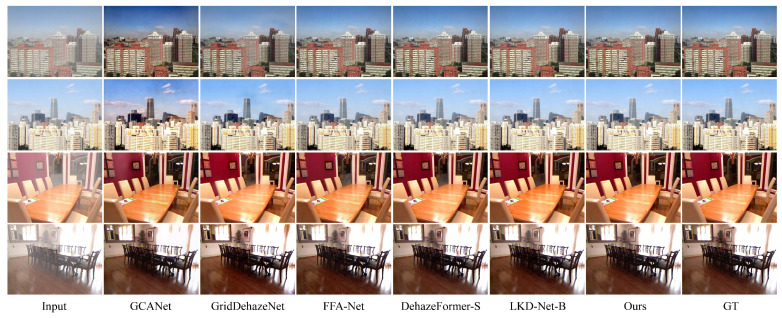
Visual comparison on the RESIDE-6K dataset. The 1st column shows the input hazy images. Columns 2 to 6 present the dehazed images generated by the comparative methods. The 7th column displays the dehazed image produced by our proposed method, and the 8th column shows the ground truth.

Two outdoor scene images and two indoor scene images were selected from the test results of the RESIDE-6K dataset to demonstrate the dehazing performance of the model. The visual comparison on the RESIDE-6K dataset is shown in Figure 4. The complexity of indoor dehazing is relatively low, as the haze usually causes lighter color distortion due to the limited light sources. In outdoor scenes, however, haze often results in severe color distortion, requiring stronger color correction and detail recovery methods during dehazing to ensure the image’s realism. As can be clearly seen from Figure 4, dehazed images processed by the GCANet and GridDehazeNet models exhibit significant color distortion, artifacts, and noise. The images processed by FFA-Net recover colors in most regions, but color distortion still exists for distant objects and small objects near the image edges. Compared to the DehazeFormer-S and LKD-Net-B models, the dehazed images processed by the model proposed in this paper more effectively restore image details and edge information, resulting in higher clarity and more natural visual effects.

**Figure 5:**
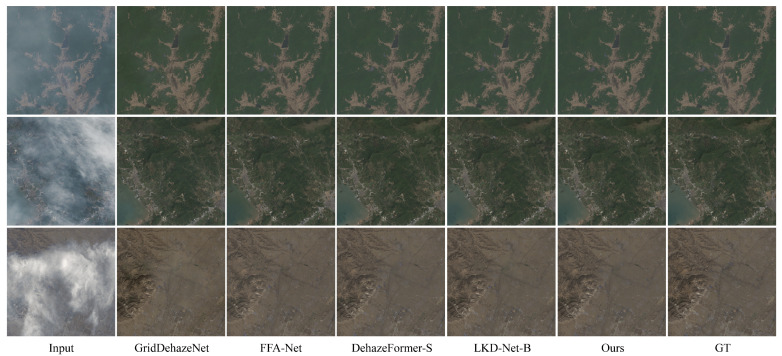
Visual comparison on the RS-Haze dataset. The 1st column presents the input hazy images. Columns 2 to 5 show the dehazed images estimated by the comparative methods. The 6th column displays the dehazed image generated by our method, and the 7th column represents the ground truth.

In the test results of the RS-Haze dataset, three images with varying haze densities from different scenes were selected to demonstrate the visual effects of different models on hazy remote sensing images. The visual comparison on the RS-Haze dataset is shown in Figure 5. After processing with the GridDehazeNet model, the remote sensing images still exhibit significant color distortion. FFA-Net, DehazeFormer-S, and LKD-Net-B are effective in removing haze under thin haze conditions but perform poorly in areas with rich texture details, where color and details cannot be fully restored. In a comprehensive comparison, the model proposed in this paper demonstrates better performance in terms of texture details, color recovery, and overall visual fidelity.

### 4.4. Ablation Study

To verify the effectiveness of the proposed model, we conducted ablation studies to systematically analyze the contributions of its components, including MF, HA, and CL-based dehazing. Furthermore, to assess the impact of data augmentation strategies in contrastive learning on dehazing performance, we categorized the image augmentations into three types: Cropping, Geometric, and Photometric. Cropping involves extracting sub-regions from the images; Geometric transformations refer to spatial modifications, such as rotation and flipping; Photometric transformations include illumination adjustment, color jittering, and Gamma correction, applied consistently to both positive and negative sample pairs. We conducted ablation experiments on these three types of augmentations to quantitatively evaluate the contribution of each strategy to the overall model performance.

**Figure 6:**
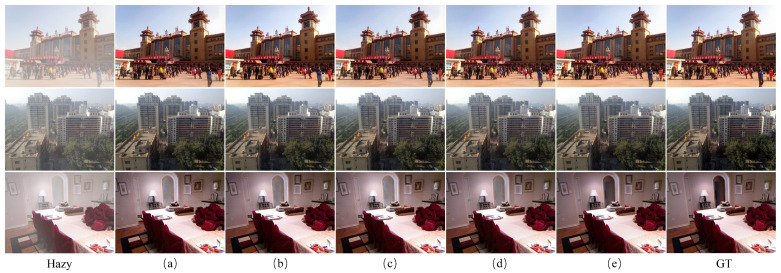
Visual comparison of ablation study results. The 1st column presents the input hazy images. Columns 2 to 6 show the dehazed images estimated by model variants (**a**–**e**) in the ablation study, respectively. The 7th column represents the ground truth.

To evaluate the contributions of each module, we first constructed a baseline network as the dehazing network, which primarily consists of two downsampling layers, two upsampling layers, and two Transformer Blocks. Subsequently, different modules were added to the baseline network in sequence to assess their respective contributions. Both training and testing were conducted on the RESIDE-6K dataset. The performance of these models is shown in Table 3. The visual comparison of these models is shown in Figure 6.

Effect of MF. MF can effectively restore high-frequency information, such as texture details, through its unique residual structure and convolutions with varying dilation rates. The seven MF modules, which progressively reduce the number of channels, can extract features at different scales and hierarchically fuse the extracted features, thereby minimizing information loss and improving the dehazing network. As shown in Table 3, MF enhances the performance of the baseline network. The PSNR of the baseline network is 28.43 dB, while the PSNR of the baseline network with MF is 30.53 dB, representing an improvement of 2.1 dB. Figure 6a shows the dehazed image estimated by the baseline network (a), which suffers from color distortion, blurred details, incomplete haze removal, and visible artifacts, resulting in overall poor visual quality. In contrast, the image estimated by the network (b), which incorporates MF, exhibits significantly improved visual performance compared to network (a). However, it still falls short in accurately restoring fine textures and preserving edge structures. The results demonstrate that the inclusion of MF can enhance the performance of the dehazing model.

Effect of HA. HA adaptively adjusts the dehazing intensity of different regions through multiple attention paths, enabling the network to more flexibly calculate region-specific weights and effectively enhancing the expressive power of features. As an auxiliary module, HA also significantly improves the dehazing performance of the model. The effect of HA can be observed by comparing network (a) and network (c). As shown in Table 3, the PSNR increases from 28.43 dB to 29.02 dB, and the SSIM rises from 0.966 to 0.968. As illustrated in Figure 6c, the network with HA effectively enhances the model’s ability to focus on key regions. Even without MF, HA can improve feature representation and image restoration by emphasizing critical features such as textures and edges while suppressing irrelevant information. The results demonstrate that the inclusion of HA enables the dehazing model to achieve better overall performance.

Effect of MF Combined with HA. The combined effect of MF and HA can be clearly observed by comparing network (b), network (c), and network (d). As shown in Table 3, network (b), which only incorporates MF, achieves a PSNR of 30.53 dB, representing a 2.1 dB improvement over the baseline network (a). Network (c), which only incorporates HA, achieves a PSNR of 29.02 dB, an increase of 0.59 dB over the baseline. When MF and HA are combined in network (d), the PSNR reaches 31.34 dB, corresponding to a 2.91 dB improvement over the baseline. Notably, this gain exceeds the sum of the individual improvements of MF and HA, indicating a synergistic effect between the two modules. As shown in Figure 6d, the network with both MF and HA not only achieves more thorough dehazing but also restores texture details more naturally. These results demonstrate that MF and HA complement each other: MF effectively extracts multi-scale features and preserves fine details, while HA emphasizes key features and suppresses irrelevant information. Their combined use enables the network to restore both local textures and global structures more effectively, leading to superior overall dehazing performance.

Effect of CL-based Dehazing. The CL-based dehazing involves constructing positive and negative sample pairs, using the InfoNCE loss function to pull anchor images towards positive samples (clear images) and push them away from negative samples (hazy images). This helps reduce the impact of haze features on the dehazing process, allowing the dehazing model to better preserve image details and structure while also improving the model’s stability when dealing with different hazy environments. Based on Table 1, the metrics of the models incorporating MF and HA in Table 3 have already surpassed most existing dehazing models. Furthermore, with the addition of the CL-based dehazing, there has been a notable breakthrough in performance, with the PSNR increasing from 31.34 dB to 31.85 dB, an improvement of 0.51 dB. From Figure 6, it is evident that, compared with the first four networks, network (e) delivers superior results in color accuracy, texture preservation, image clarity, and perceptual realism. The results demonstrate that contrastive learning not only enhances the model’s ability to represent image content through feature embeddings but also significantly improves its adaptability to varying haze densities, complex scenes, and cross-domain environments. The final images produced by the contrastive learning-based dehazing approach retain structural details while exhibiting greater stability and clarity; therefore, CL-based dehazing is an effective method for improving the performance of dehazing models.

To further evaluate the impact of data augmentation methods in contrastive learning on dehazing performance, we conducted ablation experiments on the RESIDE-6K dataset, aiming to quantitatively analyze the contribution of each augmentation strategy to the overall performance of the model. We first constructed a baseline network as the dehazing network, which did not include any data augmentation. Subsequently, different data augmentation methods were added to the baseline network in sequence to assess their individual contributions. The performance of these models is shown in Table 4.

Effect of Cropping. The ablation results presented in Table 4 indicate that cropping as a data augmentation method has a significant impact on model performance within the contrastive learning framework. The baseline model (f), which did not use any data augmentation, achieved a PSNR of 29.93 dB; after introducing cropping augmentation, the model (g) saw an increase of 0.49 dB in PSNR. The cropping operation randomly extracts local regions of the image, forcing the model to learn more discriminative local feature representations. This method increases the spatial diversity of training samples and enhances the model’s robustness. The experimental results demonstrate that, under all other conditions being equal, introducing only cropping augmentation can lead to performance improvements, confirming the effectiveness of this method in feature learning.

Effect of Geometric. The experimental results in Table 4 objectively demonstrate the impact of geometric transformation augmentation on model performance. When only cropping augmentation was applied, the model (g) achieved a PSNR of 30.42 dB. After adding geometric transformation augmentation on top of cropping, the model (h) reached a PSNR of 30.94 dB, an improvement of 0.52 dB. Geometric transformation augmentation, through operations such as rotation and scaling, expands the range of geometric variations in the training samples. This process encourages the model to learn feature representations that are stable under spatial transformations. The effectiveness of geometric transformation augmentation lies in its ability to simulate a richer set of spatial variation scenarios, thereby enhancing the model’s feature extraction capability.

Effect of Photometric. The experimental results in Table 4 objectively demonstrate the effectiveness of photometric transformation augmentation. When combined with cropping and geometric transformations, network (h) achieved a PSNR of 30.94 dB; after adding photometric transformation, network (i) reached a PSNR of 31.85 dB, yielding a gain of 0.91 dB. This improvement indicates that photometric transformation, by simulating illumination variations in real imaging conditions, can effectively enhance the model’s feature learning capability. Such augmentation enables the model to adapt to more complex visual scenes, thereby achieving higher-quality image restoration.

## 5. Conclusions

This paper presents the CL-MFHA method for image dehazing, which integrates Contrastive Learning along with a multi-scale dynamic feature fusion module and a hybrid attention mechanism. Specifically, Contrastive Learning is employed for haze-invariant representation learning by constructing positive and negative sample pairs and optimizing them using the InfoNCE loss function. This strategy effectively alleviates the negative impact of haze on feature extraction and enhances the robustness and discrimination of the learned features. The multi-scale dynamic feature fusion module focuses on separating high-frequency feature maps, thereby improving the restoration of fine image details. Working in parallel, the hybrid attention mechanism adaptively adjusts the dehazing intensity across different regions, effectively preserving texture information and improving the overall visual quality of the dehazed images. Extensive experiments conducted on standard benchmark datasets demonstrate that the proposed CL-MFHA model consistently outperforms existing state-of-the-art dehazing methods. Comparative analysis further confirms the model’s strong generalization capability across diverse dehazing scenarios, significantly enhancing both haze removal effectiveness and image detail restoration.

## Figures and Tables

**Table 1:** Comparison of different methods on the RESIDE-6K and RS-Haze datasets. The bolded values represent the optimal performance in this column.

Methods	RESIDE-6K			RS-Haze		
	PSNR (dB)	SSIM	LPIPS	PSNR (dB)	SSIM	LPIPS
GCANet [31]	26.58	0.945	0.048	34.41	0.949	0.074
GridDehazeNet [22]	25.06	0.938	0.051	34.58	0.947	0.072
FFA-Net [10]	28.32	0.953	0.032	37.40	0.955	0.060
DehazeFormer-S [13]	30.62	0.976	0.016	39.57	0.970	0.068
LKD-Net-B [32]	30.67	0.976	0.022	37.98	0.965	0.063
ConvIR-B [33]	30.96	0.966	0.015	39.47	0.963	0.062
Ours	**31.85 **	**0.980**	**0.014**	**39.76**	**0.971**	**0.059**

**Table 2:** Comparison of different methods on the NH-Haze dataset. The bolded values represent the optimal performance in this column.

Methods	PSNR (dB)	SSIM
GridDehazeNet [22]	18.33	0.667
FFA-Net [10]	19.87	0.692
DeHamer [34]	20.66	0.684
DehazeFormer [13]	20.31	0.761
DEANet [35]	20.84	0.801
OKNet [36]	20.29	0.800
Ours	**20.98 **	**0.803**

**Table 3:** Ablation study on CL-MFHA with different architectures.

Net	MF	HA	CL-Based Dehazing	PSNR (dB)	SSIM
(a)				28.43	0.966
(b)	✓			30.53	0.973
(c)		✓		29.02	0.968
(d)	✓	✓		31.34	0.976
(e)	✓	✓	✓	31.85	0.980

**Table 4:** Ablation study on data augmentation methods in contrastive learning.

Augmentation Method	Cropping	Geometric	Photometric	PSNR (dB)	SSIM
(f)				29.93	0.973
(g)	✓			30.42	0.974
(h)	✓	✓		30.94	0.976
(i)	✓	✓	✓	31.85	0.980

## Data Availability

Publicly available datasets were analyzed in this study. These data can be found at RESIDE-6K Datasets: https://www.kaggle.com/datasets/kmljts/reside-6k/data (accessed on 20 July 2025) and RS-Haze Datasets: https://www.kaggle.com/datasets/lxfbystander/rs-haze-dataset (accessed on 20 July 2025).

## Abbreviations

The following abbreviations are used in this manuscript:

CL-MFHA	Contrastive Learning-driven image dehazing with Multi-scale feature Fusion and Hybrid Attention mechanism
CL-based	Contrastive Learning-based
MF-HA-Block	Multi-scale dynamic feature Fusion and Hybrid Attention Block
MF	Multi-scale dynamic feature Fusion module
HA	Hybrid Attention mechanism
